# Glutathione-Conjugates of Deoxynivalenol in Naturally Contaminated Grain Are Primarily Linked via the Epoxide Group

**DOI:** 10.3390/toxins8110329

**Published:** 2016-11-11

**Authors:** Silvio Uhlig, Ana Stanic, Ingerd S. Hofgaard, Bernhard Kluger, Rainer Schuhmacher, Christopher O. Miles

**Affiliations:** 1 Section for Chemistry and Toxicology, Norwegian Veterinary Institute, P.O. Box 750 Sentrum, Oslo 0106, Norway; ana.stanic@vetinst.no (A.S.); chris.miles@vetinst.no (C.O.M.); 2 Department of Chemistry, University of Oslo, P.O. Box 1033, Blindern, Oslo 0315, Norway; 3 Division of Biotechnology and Plant Health, NIBIO—Norwegian Institute of Bioeconomy, Høgskoleveien 7, Ås 1430, Norway; ingerd.hofgaard@nibio.no; 4 Center for Analytical Chemistry, Department of Agrobiotechnology (IFA-Tulln), University of Natural Resources and Life Sciences, Vienna (BOKU), Tulln 3430, Austria; bernhard.kluger@boku.ac.at (B.K.); rainer.schuhmacher@boku.ac.at (R.S.)

**Keywords:** bioconjugation, HRMS, mercapturate, mycotoxin, trichothecene, thiol, LC-MS, metabolism, wheat, oats

## Abstract

A glutathione (GSH) adduct of the mycotoxin 4-deoxynivalenol (DON), together with a range of related conjugates, has recently been tentatively identified by LC-MS of DON-treated wheat spikelets. In this study, we prepared samples of DON conjugated at the 10- and 13-positions with GSH, Cys, CysGly, γ-GluCys and *N*-acetylcysteine (NAC). The mixtures of conjugates were used as standards for LC-HRMS analysis of one of the DON-treated wheat spikelet samples, as well as 19 Norwegian grain samples of spring wheat and 16 grain samples of oats that were naturally-contaminated with DON at concentrations higher than 1 mg/kg. The artificially-contaminated wheat spikelets contained conjugates of GSH, CysGly and Cys coupled at the olefinic 10-position of DON, whereas the naturally-contaminated harvest-ripe grain samples contained GSH, CysGly, Cys, and NAC coupled mainly at the 13-position on the epoxy group. The identities of the conjugates were confirmed by LC-HRMS comparison with authentic standards, oxidation to the sulfoxides with hydrogen peroxide, and examination of product-ion spectra from LC-HRMS/MS analysis. No γ-GluCys adducts of DON were detected in any of the samples. The presence of 15-*O*-acetyl-DON was demonstrated for the first time in Norwegian grain. The results indicate that a small but significant proportion of DON is metabolized via the GSH-conjugation pathway in plants. To our knowledge, this is the first report of in vivo conjugation of trichothecenes via their epoxy group, which has generally been viewed as unreactive. Because conjugation at the 13-position of DON and other trichothecenes has been shown to be irreversible, this type of conjugate may prove useful as a biomarker of exposure to DON and other 12,13-epoxytrichothecenes.

## 1. Introduction

**Figure 1:**
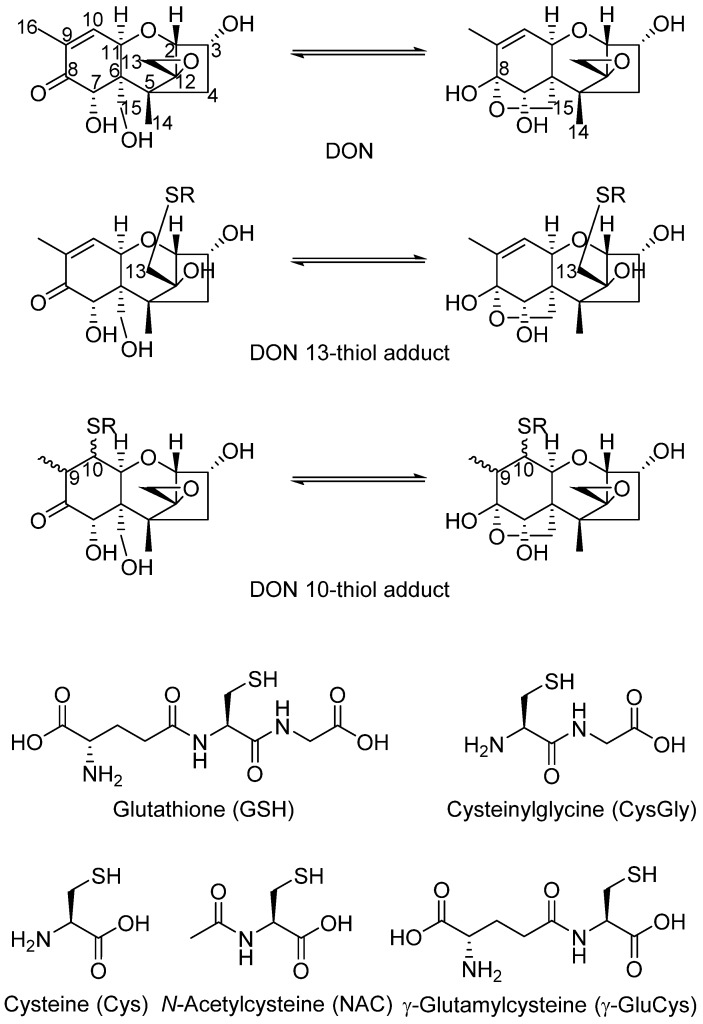
Chemical structures of 4-deoxynivalenol (DON) and its Michael (C-10) and epoxy (C-13) adducts with thiols (RSH). In solution, these compounds exist as equilibrium mixtures of their 8-keto- (left) and 8,15-hemiketal (right) forms. The structures of thiols used in this study were glutathione (GSH), cysteinylglycine (CysGly), cysteine (Cys), γ-glutamylcysteine (γ-GluCys) and *N*-acetylcysteine (NAC). Note that GSH is the tripeptide γ-GluCysGly.

The mycotoxin 4-deoxynivalenol (DON) is produced by some species of *Fusarium* that infect grain crops [1]. DON exerts a range of toxic effects in mammals and humans, e.g., on the intestine, the immune system and the brain [2,3]. The effects on the brain have been linked to the observed emesis and feed refusal in pigs [2]. Conjugation of DON with L-glutathione (GSH) was recently shown to be a biotransformation pathway in wheat plants that were injected with DON in controlled trials [4,5]. After incubation for up to 96 h, the presence of one putative DON–GSH adduct and several possible breakdown products including the cysteine (Cys) and cysteinylglycine (CysGly) adducts were tentatively identified using untargeted or targeted LC-HRMS approaches [4,5]. However, as no characterized reference standards were available, the exact structures of these adducts could not be determined. It was recently shown that thiols, including GSH, react with DON at both the C-10 and C-13 positions (Figure 1), giving rise to a range of mono- and di-conjugated products [6,7,8,9]. Because the C-8 ketone of DON is in equilibrium with the cyclic 8,15-hemiketal form, and four 9,10-diastereoisomers can be formed by thiol addition at C-10, ten mono- and eight di-conjugated toxin derivatives can be formed for any given thiol [8]. As the structures of the major thiol addition products now have been determined, and analytical standards are available [6,7], naturally produced DON–thiol adducts can be identified. Thus, the objective of this study was to use LC-HRMS methods in order to identify naturally occurring DON–thiol adducts.

## 2. Results and Discussion

### 2.1. DON–GSH and Related Adducts in DON–Treated Wheat and Naturally Contaminated Grain

**Figure 2:**
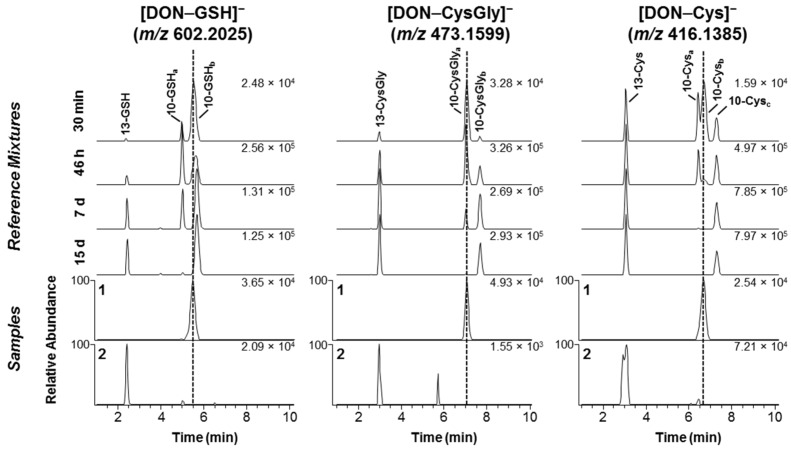
Extracted ion LC-HRMS chromatograms ([M − H]^−^, ±3 ppm) for: left, DON–GSH (*m*/*z* 602.2025); center, DON–CysGly (*m*/*z* 473.1599); and DON–Cys (*m*/*z* 416.1385). For each sample, the upper four stacked chromatograms are from the mixture of DON, GSH, CysGly and Cys (pH 10.7) at various reaction times. The two lower chromatograms are from the extract of wheat that was treated with deoxynivalenol for 96 h (**1**), and from an extract of naturally-contaminated Norwegian oats (**2**). Epoxide conjugates (addition at C-13) eluted at 2–3 min, whereas the Michael conjugates (addition at C-10) eluted at ca. 4.5–7 min. The number in the top right-hand corner of each chromatogram is the intensity of the highest peak in that chromatogram (arbitrary units). Subscripts (a–c) indicate different diastereoisomers).

When wheat flowering ears were treated with DON and then extracted and analyzed after 12, 24, 48 and 96 h post-treatment, several putative DON conjugates, including DON–GSH and related derivatives, were detected as early as 12 h post-injection [4,5]. Although the LC-HRMS and LC-HRMS/MS data showed that these products were conjugates of DON, the approach used did not facilitate the detailed elucidation of the conjugation site, type of linkage and isomer that had been formed during the incubation. We therefore used LC-HRMS to compare an extract from a DON-treated wheat plant (*Fusarium*-head-blight-susceptible cultivar “Remus”, 96 h post-exposure) with synthetic reaction mixtures containing different ratios of the DON-10- and DON-13-conjugates of GSH, CysGly, γ-GluCys and Cys (Figure 2 and Figure S1). The chromatogram from the extract of the DON-treated wheat showed one prominent peak corresponding to one of the C-10 conjugates for each of DON–GSH, DON–CysGly and DON–Cys as had already been suggested by the authors of the earlier studies [4,5]. The type of conjugate present in the extract corresponded to a kinetically favored, and thus relatively fast-forming, isomer (see chromatogram **1** for DON–GSH, –CysGly, and –Cys in Figure 2) from Michael addition at C-10 (Figure 1). It has been shown that this initial product from the reaction of DON with mercaptoethanol, Cys or GSH is replaced (under neutral or mildly basic reaction conditions) by other C-10 conjugates and a C-13 conjugate over time [6,7,8], and the same occurred in the present study during conjugation with GSH, CysGly, Cys (Figure 2), γ-GluCys and *N*-acetylcysteine (NAC) (Figure S1).

**Figure 3:**
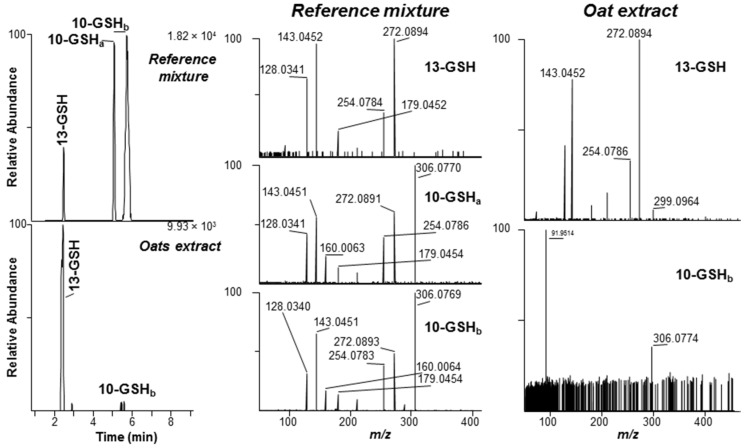
Left-hand panels, extracted ion LC-HRMS/MS chromatograms (*m/z* 272.0894 + 306.0769; ±5 ppm) precursor ions at *m*/*z* 602.2 ([M − H]^−^ for DON–GSH) of: a mixture of DON, GSH, CysGly, γ-GluCys and Cys (pH 10.7) after reaction for one week (upper chromatogram); and an extract from naturally-contaminated ripe Norwegian oat grains (lower chromatogram). The early-eluting adduct is DON-13-GSH (from addition of l-glutathione to the epoxide group of DON), while the later-eluting adducts are from isomers of DON-10-GSH (from addition to the 9,10-double bond of DON). Comparison of the HRMS/MS spectrum of the early-eluting peak from the oats (right-hand upper panel) with that of DON-13-GSH in the reaction mixture (center upper panel) confirmed its identity. The *m/z* 306.0774 product-ion in the later-eluting conjugate in the oats (lower right-hand panel) suggested that the oats also contained minor amounts of DON-10-GSH, conjugate since this was also the most prominent product-ion in all the semi-synthetic isomers of DON-10-GSH (lower two panels for the reference mixture). Subscripts (a,b) indicate different diastereoisomers.

In contrast to the DON-treated wheat ear sample, which had been sampled at the flowering stage, the 35 naturally-contaminated grain samples were all harvested at the end of the growing season. Thus, these plants had potentially been exposed to DON for much longer than the artificially-contaminated spring wheat (96 h). Of the 35 grain samples, 22 were found to contain at least one DON–thiol conjugate. DON–GSH conjugates were both most prevalent (found in all the 22 DON–thiol containing samples), and in general detected at the highest relative concentration based on relative peak areas, of the thiol-conjugates detected (Table S1). DON–CysGly and DON–Cys conjugates were found in two and 11 of the 35 grain samples, respectively. A few of the samples appeared to contain low levels of the same rapidly-forming diastereoisomer of the C-10 adducts of GSH, CysGly and Cys that were observed in the DON-treated wheat (Figure 2). However, the dominant isomer in the naturally-contaminated grain samples of both oats as well as wheat was the early-eluting C-13 conjugate, which was supported by comparing the LC-HRMS chromatograms with those from the synthetic reaction mixtures (Figure 2) and authentic standards of DON-13-GSH and DON-13-Cys. This finding was verified by HRMS/MS targeting the [M − H]^−^ ions of DON–GSH (Figure 3). We previously reported that MS-fragmentation of the [M − H]^−^ ions of DON–thiol conjugates is well suited to distinguishing between the products from Michael addition to C-10 and addition to the epoxide at C-13 [6,7]. Thus, fragmentation of the C-10 conjugates yielded primarily negatively charged amino acid or peptide fragments, while fragmentation of the C-13 conjugates gave product ions primarily related to the trichothecene moiety [6,7]. A concentrated (approximately 5:1) oat extract was used to obtain HRMS/MS spectra of the DON–GSH isomers present in the sample, and the resulting HRMS/MS spectrum of the presumed DON-13-GSH conjugate in the oats was essentially identical to the HRMS/MS spectrum of DON-13-GSH in the reference mixture (Figure 3). The signal/noise of the [M − H]^−^ ions for the DON-10-GSH conjugate in the sample was low, and only the major product ion from targeted-HRMS/MS was observed (*m/z* 306.0774 for [GSH−H]^−^, Δ −2.9 ppm) (Figure 3).

**Figure 4:**
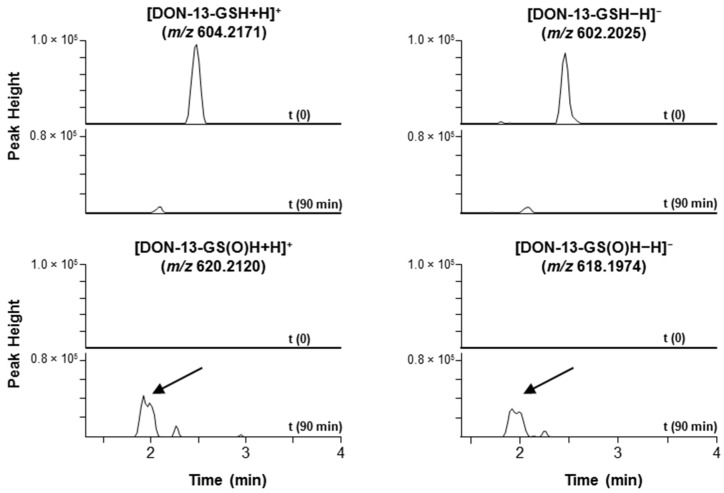
Extracted ion LC-HRMS chromatograms (±5 or 7.5 ppm for positive/negative ion mode, respectively) for [M + H]^+^ and [M − H]^−^ of DON-13-GSH and its sulfoxide, DON-13-GS(O)H, in an extract of ripe Norwegian oat grains. The upper two chromatograms show the disappearance of the DON-13-GSH peak after treatment of the extract with hydrogen peroxide for 90 min, while the lower two chromatograms show the concurrent appearance of the partially separated peaks from a pair of DON-13-GS(O)H diastereoisomers.

Sulfides are known to be oxidized by 30% hydrogen peroxide to sulfoxides or sulfones [10,11], and thiol-conjugates of DON are conveniently oxidized to their sulfoxides using this approach without further oxidation to sulfones [6,8]. Oxidation with hydrogen peroxide followed by LC-MS analysis may thus be applied as a simple technique to verify putative DON–thiol conjugates in complex mixtures, an approach analogous to that developed for identifying Met-containing peptides in cyanobacterial blooms [12]. Addition of hydrogen peroxide to an extract from Norwegian oats oxidized approximately 90% of the DON-13-GSH to the corresponding sulfoxides (DON-13-GS(O)H) within 90 min (Figure 4). This resulted in a decrease in the intensities of the [M + H]^+^ and [M − H]^−^ peaks for DON-13-GSH, and the concurrent appearance of two major peaks for the diasteroisomers of DON–GS(O)H at *m/z* 620.2137 ([M + H]^+^, Δ2.8 ppm) and 618.2014 ([M − H]^−^, Δ6.4 ppm) (Figure 4). Similar results were obtained when an aliquot of a mixture from reaction of DON with GSH was treated with hydrogen peroxide for 60 min (Figure S2).

The composition of DON–thiol conjugates in the DON-injected wheat sample and the naturally-contaminated oat and wheat samples are in accord with observations from earlier studies on the complex and dynamic reaction of DON with various thiols [6,7,8]. The epoxide conjugates form irreversibly but relatively slowly, while the diastereomeric Michael addition products form reversibly with different, but generally much faster, reaction rates [6,7,8]. This is therefore consistent with finding the most rapidly-formed C-10 Michael adduct in the DON-injected wheat, but predominantly the corresponding C-13 epoxide adducts in the naturally-contaminated grain that potentially had been exposed to DON over a much longer time. Together with the fact that *Fusarium graminearum* is known to produce DON as a virulence factor of fungal spread already during the early stage of host infection, these findings also suggest that the DON-13-thiol conjugates detected in naturally-contaminated harvest-ripe grain might be largely the result of chemical, rather than enzymatic, reactions. If the formation of DON-13-thiol conjugates was an enzyme-catalyzed reaction it would be expected to observe such conjugates in the sample from the DON-treated wheat spikelet. In vitro, conjugation of DON with thiols generally proceeds to give primarily Michael adducts initially, but the reactions often do not go to completion due to autoxidation of the thiol [6,7,8]. However, in vivo produced DON would be exposed continuously to consistent endogenous physiological concentrations of GSH that would allow the slow but irreversibly-formed DON-13-GSH to accumulate.

**Figure 5:**
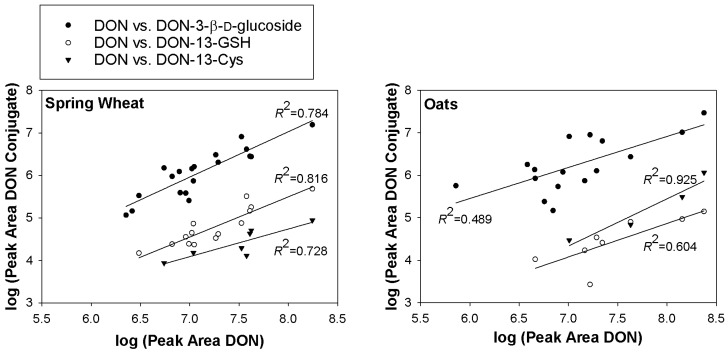
Plots of the log–transformed LC-HRMS peak areas of DON ([M + formate]^−^) versus those for DON-3-β-D-glucoside ([M + formate]^−^), DON-13-GSH ([M − H]^−^), and DON-13-Cys ([M − H]^−^) in extracts from naturally contaminated Norwegian spring wheat (left) and oats (right). Curves and squared correlation coefficients are from least squares regression.

The peak areas for DON-13-GSH and DON-13-Cys in the LC-HRMS chromatograms from the grain samples correlated well with those of DON (Figure 5). However, the peak areas of the conjugates were typically 100–1000-fold lower than that of DON in the same sample. The peak area of DON-3-β-D-glucoside likewise also correlated well with the peak area of DON (Figure 5).

### 2.2. Mercapturates of DON

**Figure 6:**
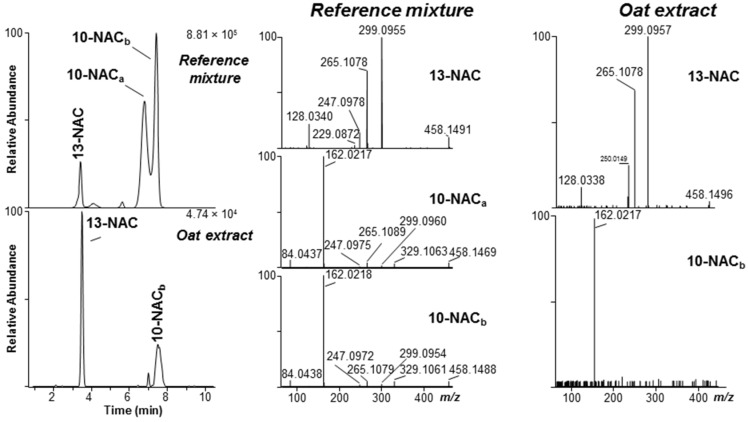
Left, extracted ion LC-HRMS chromatograms ([M − H]^−^, ±3 ppm) for DON–NAC adducts (*m*/*z* 458.1490) (“DON mercapturates”) in a semi-synthetic reference mixture (upper), and in an extract from ripe naturally-contaminated Norwegian oat grains (lower). Comparison of the HRMS/MS spectra of the [M − H]^−^ ions (*m/z* 458.15) in the reference mixture with those in the grain extract supported the identity of compounds in oats as being DON-13-NAC (early-eluting conjugate) and DON-10-NAC (later-eluting conjugate). Subscripts (a,b) indicate different diastereoisomers.

**Figure 7:**
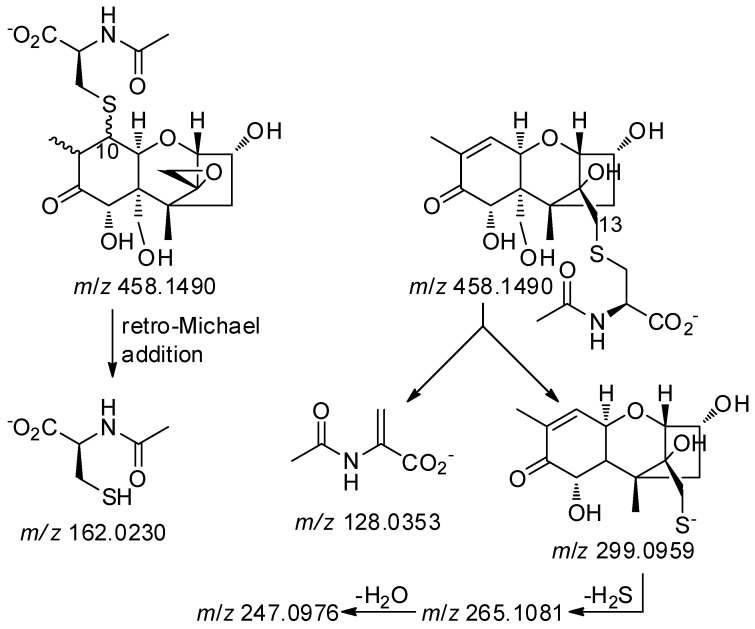
Major product ions observed during fragmentation of [M − H]^−^ for DON–NAC adducts, and their proposed origins. The negative charge is arbitrarily depicted on the most acidic position in the fragments.

In plants, GSH conjugates of xenobiotics are thought to be deposited in the vacuoles [13], where they can be degraded enzymatically to CysGly and Cys conjugates [14]. However, the metabolic fate of the resulting cysteine conjugates is less well established. In plants, the resulting Cys-conjugates can be *N*-acetylated, or *N*-malonylated followed by decarboxylation, to form *N*-acetylcysteine (NAC) conjugates (mercapturates) [15]; while in mammals they could also be cleaved by cysteine *S*-conjugate β-lyase (usually present in liver and kidneys) to mercaptans [16]. Although NAC biotransformation products are rarely reported in plants, we specifically looked for DON–NAC conjugates in extracts from naturally DON-contaminated grain and the DON-injected wheat by plotting the appropriate LC-HRMS extracted ion chromatograms (Figure 6). We then compared the chromatograms with those from the reaction of DON with NAC (Figure 6). As with DON–GSH, –CysGly, –γ-GluCys and –Cys conjugates, the DON—NAC conjugates afforded both [M + H]^+^ (*m/z* 460.1641, Δ1.1 ppm) and [M − H]^−^ (*m/z* 458.1515–458.1517, Δ 5.4–5.8 ppm) ions upon electrospray ionization. However, the signal/noise of extracted ion chromatograms from the naturally-contaminated grain samples was higher in the chromatograms from negative ionization. Where detected, the isomer-profile of the putative DON–NAC conjugates in the grain resembled those observed for the other DON–thiol conjugates in grain, with one major early-eluting conjugate consistent with addition to C-13, and another minor later-eluting conjugate consistent with addition to C-10. DON–NAC conjugates were not detected in the DON-injected wheat sample. The identity of the conjugates was confirmed by comparing product-ion spectra from targeted LC-HRMS/MS of the [M − H]^−^ ions of the products from reaction of DON with NAC with those of the putative DON–NAC conjugates in a concentrated oat extract (Figure 6). Both the retention time and the HRMS/MS spectrum of [M − H]^−^ for the putative DON-13-NAC in the oat extract were essentially identical to those of DON-13-NAC in the reference mixture (Figure 6). The base peak in the HRMS/MS spectrum of DON-13-NAC was at *m/z* 299.0955–299.0957 (C_14_H_19_O_5_S^−^, Δ−1.2 to −0.6 ppm) (Figure 6). This product-ion has been observed for other DON-13-thiol conjugates and can be attributed to cleavage of the S–C bond on the amino-acid-side of the sulfide bond with concomitant loss of CH_2_O from C-6 (Figure 7) [6]. On the other hand, the base peak in the HRMS/MS spectrum of the putative DON-10-NAC conjugate was at *m/z* 162.0217–162.0218 (C_5_H_8_O_3_NS^−^, Δ−8.3 to −7.6 ppm), corresponding to deprotonated NAC (Figure 6 and Figure 7) produced by retro-Michael addition. Thus, the above data confirm the presence of DON-13-NAC and DON-10-NAC in Norwegian grain.

### 2.3. Separation of DON–Thiol Adducts

In earlier LC-MS studies of thiol-conjugation of DON, it was challenging to separate the isomeric reaction products on an octadecylsilane stationary phase [6,8]. Pentafluorophenylpropyl (PFPP) phases have recently shown to provide unique selectivity, especially for separation of regio- and stereo-isomers [17]. We used a PFPP column with core-shell particles that provided improved separation of the DON–GSH conjugates [7]. Up to three of the four possible 9,10-diastereoisomers of DON-10-Cys and DON-10-NAC could be separated, while only two baseline-separated peaks were obtained for DON-10-GSH, DON-10-CysGly and DON-10-γ-GluCys (Figure 2 and Figure S1). However, we have earlier shown that the broader later-eluting peak of DON-10-GSH contained at least two closely-eluting isomers with small retention time differences but marked differences in positive ion HRMS/MS spectra [7]. Negative ion HRMS/MS spectra did not discriminate between these isomers of DON-10-GSH [7], as was also observed in the present study (Figure 3). Similar broadened peaks were also observed for DON-10-GSH (*t*_R_ 5.5 min), DON-10-CysGly (*t*_R_ 7.0 min), DON-10-γ-GluCys (*t*_R_ 4.7 min), DON-10-Cys (*t*_R_ 6.5 min) and DON-10-NAC (*t*_R_ 6.7 min) in negative ion LC-MS (Figure 2 and Figure S1).

### 2.4. DON–Acetates

**Figure 8:**
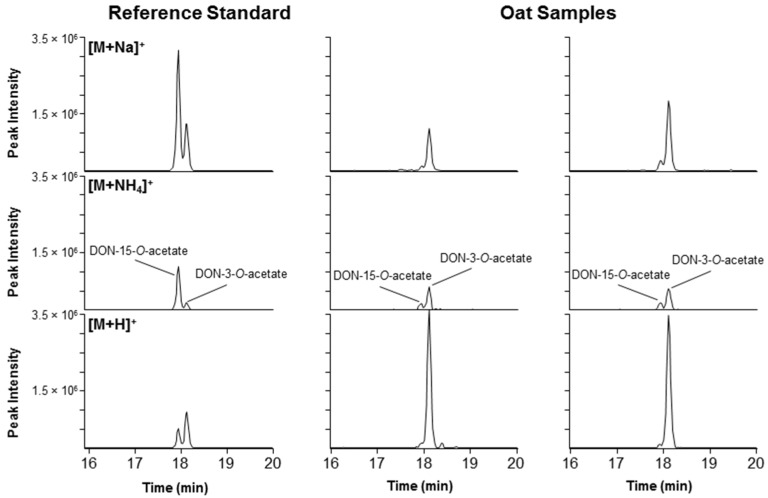
Extracted ion chromatograms (±3 ppm) from LC-HRMS showing the separation and concurrent presence of DON-3-*O*-acetate and DON-15-*O*-acetate in grain. Left, a mixed standard containing 50 ng/mL of both acetates; middle and right, extracts from two naturally-contaminated Norwegian oat samples. The top, middle and lower panels show the extracted ion chromatograms at *m/z* 361.1258 [M + Na]^+^, 356.1704 [M + NH_4_]^+^, and 339.1438 [M + H]^+^, respectively. The relatively higher peak intensities of [M + H]^+^ for DON-3-*O*-acetate in the samples vs. the reference standard could be due to matrix signal enhancement.

Several of the grain samples contained both DON-3-*O*-acetate and DON-15-*O*-acetate, with DON-3-*O*-acetate as the dominant analogue (Figure 8). The chromatographic separation of the two isomers has rarely been shown but was recently achieved using a similar HPLC column chemistry (pentafluorophenyl) as was used in this study for separation of DON–thiol isomers [18]. While DON-3-*O*-acetate is a common contaminant of cereal grain in Norway [19], this is the first report of DON-15-*O*-acetate in Norwegian grain and corresponds well with the DON-15-*O*-acetate producing genotypes of *Fusarium graminearum* observed for the first time in Norway in 2006 [20]. The identity of the two DON-acetates was supported by comparison of the LC-HRMS/MS spectra of the [M + H]^+^ ions in the reference standard and the oat extract (Figure S3).

## 3. Conclusions

This is the first report of the natural occurrence of 10- and 13-conjugates of DON with GSH, CysGly, Cys and NAC. The finding of NAC-conjugates is of special interest since such biotransformation products are rarely reported in plants. We did not aim to quantify the concentrations of DON–thiol conjugates. However, our experience regarding relative responses of different types of molecules in the used LC-HRMS instrument suggests that the concentrations of the DON–thiol conjugates in the samples were substantially lower than those of DON itself or DON-3-glucoside. The origin of the DON–NAC conjugates remains to be elucidated, i.e., if they are the result of acetylation of DON–Cys or the product of decarboxylation of a possible DON-malonylcysteine conjugate. Another question that needs to be addressed is the involvement of glutathione *S*-transferases in the conjugation in plants. Our results suggest that at least the formation of DON-13-GSH is merely the result of chemical reaction rather than due to enzyme-catalyzed conjugation. Since DON-13-GSH is likely to be nontoxic and its formation irreversible, the identification of cereal genotypes that utilize a detoxification pathway that leads to such a product may prove useful in future breeding strategies aiming to reduce DON accumulation in cereals.. Furthermore, the presence of the sulfur-linked DON conjugates suggests that other plant-derived thiols, such as cysteine-containing proteins, also may bind to DON. The latter suggest a possible use of such conjugates as biomarkers.

## 4. Materials and Methods

### 4.1. Chemicals and Reagents

DON (≥98%), GSH (≥98%), Cys, γ-GluCys, CysGly, NAC and Na_2_CO_3_ (pro analysis) were from Sigma-Aldrich (Steinheim, Germany), and NaHCO_3_ (pro analysis) was from Fluka (Steinheim, Germany). NaHCO_3_ and Na_2_CO_3_ were used to prepare 0.2 M buffer with pH of 10.7 as measured with a Mettler Delta 320 pH meter at ambient temperature. Purified quantitative standards of DON-10-Cys, DON-10-GSH, DON-13-Cys and DON-13-GSH were available from previous work [6,7], while DON-3-O-β-d-glucoside, DON-3-*O*-acetate and DON-15-*O*-acetate were from Romer Labs (Tulln, Austria).

### 4.2. Preparation of Reference Standard Mixtures

DON (100 μg) was dissolved in 1 mL of a freshly prepared solution of GSH, CysGly, γ-GluCys and Cys (10 mM each) in 0.2 M carbonate buffer (pH 10.7). Aliquots (20 μL) were periodically transferred to chromatography vials, diluted to 1 mL with water, and 1.5 μL acetic acid was added to stop the reaction. Separate reference mixtures were prepared by dissolving 100 μg DON in 1 mL 10 mM NAC in 0.2 M carbonate buffer (pH 10.7). Aliquots were prepared in the same way as for the mixture containing the four thiols above.

### 4.3. Samples and Extraction

Milled samples of naturally DON-contaminated grains of Norwegian spring wheat and oats were obtained from the Norwegian Institute of Bioeconomy Research, NIBIO [21]. The samples were from the 2004 to 2011 growing seasons, and were selected because of their relatively high DON contamination (1100–11,000 μg/kg). The grain samples had been milled after harvest and stored at −20 °C thereafter. The DON concentrations in these samples had been determined by LC-MS/MS in earlier projects [21]. The presence of DON in these samples was verified in all samples (Table S1), but we did not make efforts to re-quantify the toxin in this study. Aliquots of 0.1 g were weighed into 1.5 mL Eppendorf tubes and 1 mL methanol/water (3:1, *v/v*), containing 0.1% formic acid, was added. The samples were vortex-mixed for ca. 10 s, and then placed in an ultrasonic bath for 15 min followed by centrifugation at 15,000 *g* for 5 min. Supernatants were filtered through 0.45 μm PTFE syringe filters (Phenomenex) and transferred to chromatography vials and sealed.

An extract from DON-treated spikelets of spring wheat cultivar “Remus”, which is highly susceptible to *Fusarium* head blight, was obtained from the IFA-Tulln, Austria. The plant ears had been treated with 1 mg DON/wheat ear in a greenhouse experiment. Treated spikelets had been sampled and extracted after incubation for 96 h [4]. The extraction protocol was identical to that used for extraction of the naturally-contaminated grain samples.

### 4.4. LC-HRMS(/MS) Analyses

Separation was achieved using a 150 × 2.1 mm, i.d. 2.6 μm Kinetex F5 column (Phenomenex, Torrance, CA, USA). Injection volumes were 1–5 μL. The mobile phase (250 μL/min) consisted of 5 mM ammonium formate (A), and 5 mM ammonium formate in 95:5 methanol–water (B), in a linear gradient from 3%–40% B over 14.5 min, then to 100% B at 14.7 min (2-min hold), followed by return to 3% B at 16.9 min, and equilibration with 3% B for 3.1 min using a Dionex UltiMate 3000 UPLC pump (Thermo Fischer Scientific, Waltham, MA, USA). The detector was a Q-Exactive Fourier-transform high-resolution mass spectrometer (Thermo Fischer Scientific) equipped with a heated electrospray ionization interface. The HRMS was run in positive and negative ion full-scan mode using fast polarity switching (i.e., alternating positive and negative ion scans), in the mass range *m/z* 150–1200 (Table 1). The mass resolution was set to 70,000 at *m/z* 200. The spray voltage was 4 kV, the transfer capillary temperature was 250 °C, and the sheath and auxiliary gas flow rates were 35 and 10 units, respectively. Exact values of *m/z* used for extracted ion LC-HRMS chromatograms for DON acetates, thiol conjugates and their sulfoxides (Table 1) were in this study obtained using Fusarium toxin mass calculator version 9 [22], while Xcalibur 2.3 was used to calculate the errors in the accurate masses.

LC-HRMS/MS was used to acquire high-resolution MS/MS spectra for selected DON–thiol conjugates in a concentrated oat sample and compared to those obtained from semi-synthetic reference compounds, using the same chromatographic conditions and detector as above. Analyses were performed in negative mode, using targeted MS/MS scans. The precursor ion mass for the MS/MS spectra was selected with a quadrupole isolation window of *m/z* 2.0. The normalized collision energy for higher-energy collisional dissociation was set to 30 and the resolution during product ion scanning was set to 17,500 or 35,000.

### 4.5. Oxidation of DON–GSH to DON–GS(O)H with Hydrogen Peroxide

Hydrogen peroxide (30%, 15 μL) was added to a 100 μL aliquot of a methanol–water extract from oats in a sample vial and sealed. The same amount was also added to a 100 μL aliquot of the acidified reference mixture (*t* = 1 week) from reaction of DON with GSH, CysGly, γ-GluCys and Cys. Oxidation of DON–GSH to its sulfoxide, DON–GS(O)H, was shown by injections into the LC-HRMS system just before addition of hydrogen peroxide and after a reaction time of 90 min (20 °C). The signal/noise for other DON–thiols in the sample was too low to obtain clear data for products from sulfide oxidation.

## Figures and Tables

**Table 1:** Exact values of *m/z* for ions of DON and its derivatives used in this study [22].

Compound	Ion Species ESI^+^	Calculated Mass (*m/z*)	Ion Species ESI^−^	Calculated Mass (*m/z*)
Deoxynivalenol (DON)	-	-	[M + formate]^−^	341.1242
3/15-*O*-acetyl-DON	[M + H]^+^	339.1438	-	-
	[M + NH_4_]^+^	356.1704		
	[M + Na]^+^	361.1258		
DON–GSH	[M + H]^+^	604.2171	[M − H]^−^	602.2025
DON–GS(O)H	[M + H]^+^	620.2120	[M − H]^−^	618.1974
DON–CysGly	[M + H]^+^	475.1745	[M − H]^−^	473.1599
DON–γ-GluCys	[M + H]^+^	547.1956	[M − H]^−^	545.1811
DON–Cys	[M + H]^+^	418.1530	[M − H]^−^	416.1385
DON–NAC	[M + H]^+^	460.1636	[M − H]^−^	458.1490
DON-3-β-d-glucoside	[M + NH_4_]^+^	476.2126	[M + formate]^−^	503.1770

## Supplementary Materials

The following are available online at www.mdpi.com/2072-6651/8/11/329/s1, Table S1: Samples and individual peak areas, Figure S1: Extracted ion chromatograms for products from reaction of DON with γ-GluCys and NAC, Figure S2: *S*-oxidation of DON–GSH reference mixture, Figure S3: Product ion spectra of DON–acetates in reference standard and an oats sample.

## Abbreviations

The following abbreviations are used in this manuscript:

Cys	l-cysteine
DON	4-deoxynivalenol
γ-GluCys	γ-l-glutamyl-l-cysteine
CysGly	l-cysteinyl-l-glycine
GSH	l-glutathione
HRMS	high-resolution mass spectrometry
LC	liquid chromatography
MS/MS	tandem mass spectrometry
NAC	*N*-acetylcysteine
PFPP	pentafluorophenylpropyl
*t*_R_	retention time
