# Supplementary material for: Glutathione-Conjugates of Deoxynivalenol in Naturally Contaminated Grain Are Primarily Linked via the Epoxide Group
Source: Toxins (Basel). 2016 Nov 11;8(11):329. doi: 10.3390/toxins8110329 (PMC5127126; doi:10.3390/toxins8110329)
Supplement: Supplementary file 1 [file toxins-08-00329-s001.pdf]

Supplementary Materials: Glutathione-Conjugates of Deoxynivalenol in Naturally Contaminated Grain are Primarily Linked via the Epoxide Group

Silvio Uhlig, Ana Stanic, Ingerd S. Hofgaard, Bernhard Kluger, Rainer Schuhmacher and Christopher O. Miles

Table S1. List of samples and peak areas from LC–HRMS extracted ion chromatograms (±5 ppm).

Original Sample Number	Grain Species	Year	DON [1] (mg/kg)	Peak Area, [DON + formate]$^-$	Peak Area, [DON-13-GSH – H]$^-$	Peak Area, [DON-13-CysGly – H]$^-$	Peak Area, [DON-13-Cys – H]$^-$	Peak Area, [DON-13-NAC – H]$^-$	Peak Area, [DON-10-GSH – H]$^-$	Peak Area, [DON-10-Cys – H]$^-$	Peak Area, [DON-10-NAC – H]$^-$
217	Spring wheat	2004	1.4	7.8×10^6	-	-	-	-	-	-	-
25 *	Spring wheat	2006	1.8	3.3×10^7	7.5×10^4	-	2.0×10^4	1.6×10^5	-	-	-
697	Spring wheat	2006	1.4	5.5×10^6	-	-	8.7×10^3	-	-	-	-
111	Spring wheat	2006	2.4	1.1×10^7	7.3×10^4	-	1.5×10^4	-	-	-	-
30	Spring wheat	2006	3.7	1.8×10^7	3.3×10^4	-	-	-	-	-	-
137	Spring wheat	2006	1.1	1.1×10^7	4.4×10^4	-	-	-	-	-	-
50	Spring wheat	2006	1.5	3.1×10^6	1.5×10^4	-	-	-	-	-	-
817 *	Spring wheat	2007	1.1	1.9×10^7	4.2×10^4	-	-	6.0×10^4	-	-	6.1×10^3
795 *	Spring wheat	2007	8.7	1.8×10^8	4.8×10^5	-	8.8×10^4	1.4×10^5	8.4×10^4	4.1×10^4	3.5×10^4
1236	Spring wheat	2008	1.2	2.6×10^6	-	-	-	-	-	-	-
1296	Spring wheat	2008	1.2	8.0×10^6	-	-	-	-	-	-	-
1435 *	Spring wheat	2009	2.2	3.8×10^7	3.2×10^5	-	1.3×10^4	9.7×10^4	5.0×10^4	-	-
1424	Spring wheat	2009	1.8	1.1×10^7	2.3×10^4	-	-	-	-	-	-
1503 *	Spring wheat	2010	1.6	4.2×10^7	1.8×10^5	-	5.1×10^4	1.3×10^5	-	-	-
1516 *	Spring wheat	2010	5.7	4.1×10^7	1.5×10^5	-	4.4×10^4	1.3×10^5	-	-	-
1780	Spring wheat	2011	1.4	2.3×10^6	-	-	-	-	-	-	-
1772	Spring wheat	2011	1.3	6.6×10^6	2.4×10^4	-	-	-	-	-	-
1787	Spring wheat	2011	2.4	9.1×10^6	3.6×10^4	-	-	-	-	-	-
1799	Spring wheat	2011	2.8	9.8×10^6	2.4×10^4	-	-	-	-	-	-
243	Oats	2004	1.7	7.8×10^6	-	-	-	-	-	-	-
510	Oats	2005	1.7	2.2×10^7	2.6×10^4	-	-	-	-	-	-
713 *	Oats	2006	8.8	2.4×10^8	1.4×10^5	1.9 × 104	1.2×10^6	1.4×10^5	-	5.0×10^4	-
708 *	Oats	2006	6.4	1.4×10^8	9.2×10^4	7.9 × 103	3.1×10^5	1.1×10^5	-	-	-
699	Oats	2006	2.1	1.0×10^7	-	-	3.0×10^4	-	-	-	-
816	Oats	2007	3.3	4.6×10^6	1.1×10^4	-	-	-	-	-	-
788	Oats	2007	5.5	4.0×10^7	8.0×10^4	-	6.9×10^4	2.1×10^4	-	-	-
1228	Oats	2008	2.1	1.7×10^7	2.7×10^3	-	-	-	1.0×10^4	-	-
1153	Oats	2008	1.9	7.2×10^5	-	-	-	-	-	-	-
1197	Oats	2008	3.4	1.5×10^7	1.7×10^4	-	-	-	4.5×10^3	-	-
1309	Oats	2008	8.9	4.5×10^6	-	-	-	-	-	-	-
1419	Oats	2009	2.1	8.8×10^6	-	-	-	-	-	-	-

Table s1. *Cont.*

Original Sample Number	Grain Species	Year	DON [1] (mg/kg)	Peak Area, [DON + formate]⁻	Peak Area, [DON-13-GSH − H]⁻	Peak Area, [DON-13-CysGly − H]⁻	Peak Area, [DON-13-Cys − H]⁻	Peak Area, [DON-13-NAC − H]⁻	Peak Area, [DON-10-GSH − H]⁻	Peak Area, [DON-10-Cys − H]⁻	Peak Area, [DON-10-NAC − H]⁻
1625	Oats	2010	3.3	1.9×10^7	3.4×10^4	-	-	-	-	-	-
1471	Oats	2010	1.1	3.8×10^6	-	-	-	-	-	-	-
1541	Oats	2010	1.7	5.7×10^6	-	-	-	-	-	-	-
1555	Oats	2010	1.9	7.0×10^6	-	-	-	-	-	-	-

[1] Concentrations are from earlier work; DON was not quantified in this study; * Samples were concentrated (5×) in order to obtain HRMS/MS data for NAC-conjugates.

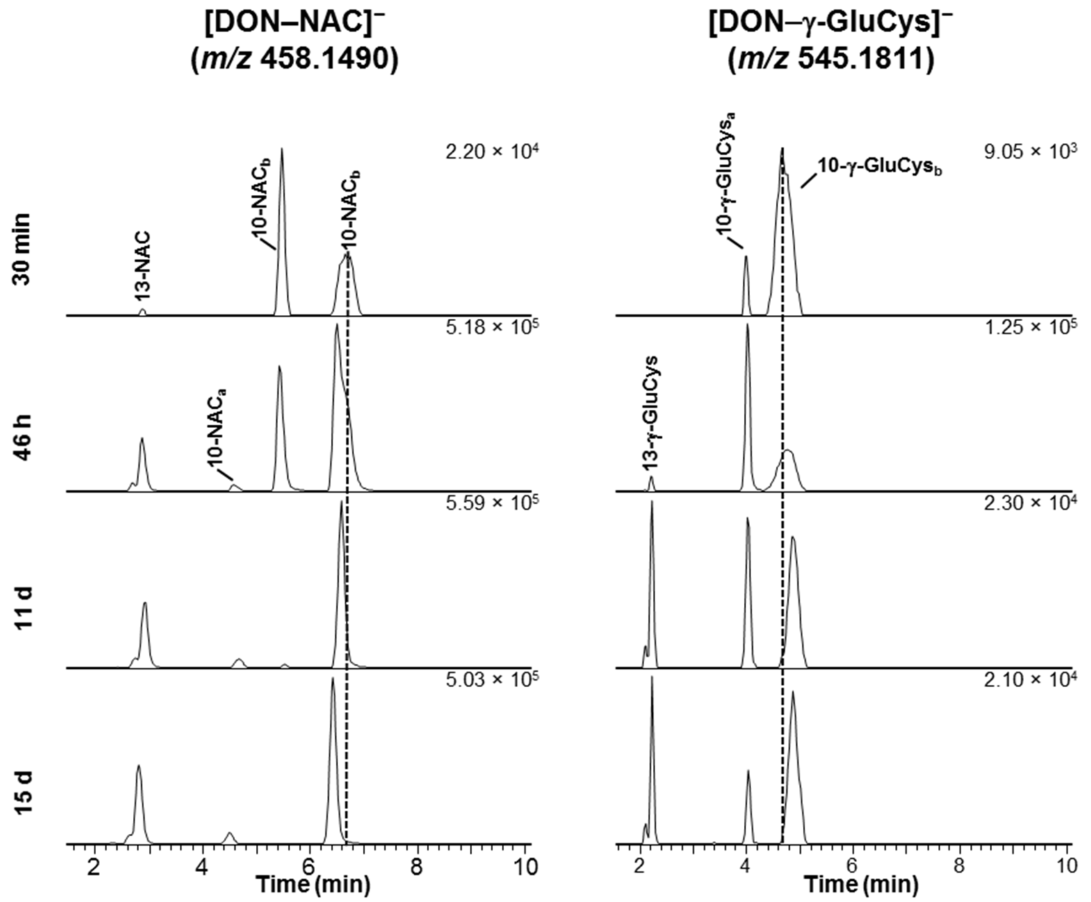

Figure S1. Extracted ion LC-HRMS chromatograms ($[M - H]^-$, ±5 ppm) for, left, DON–NAC (*m*/*z* 458.1490); right, DON–γ-GluCys (*m*/*z* 545.1811). The four stacked chromatograms are from a mixture of DON with the corresponding thiol (pH 10.7) at various reaction times. Epoxide conjugates (addition at C-13) eluted at 2–3 min, whereas the Michael conjugates (addition at C-10) eluted at ca 4.5–7 min. The number in the top right-hand corner of each chromatogram is the intensity of the highest peak in that chromatogram (arbitrary units)

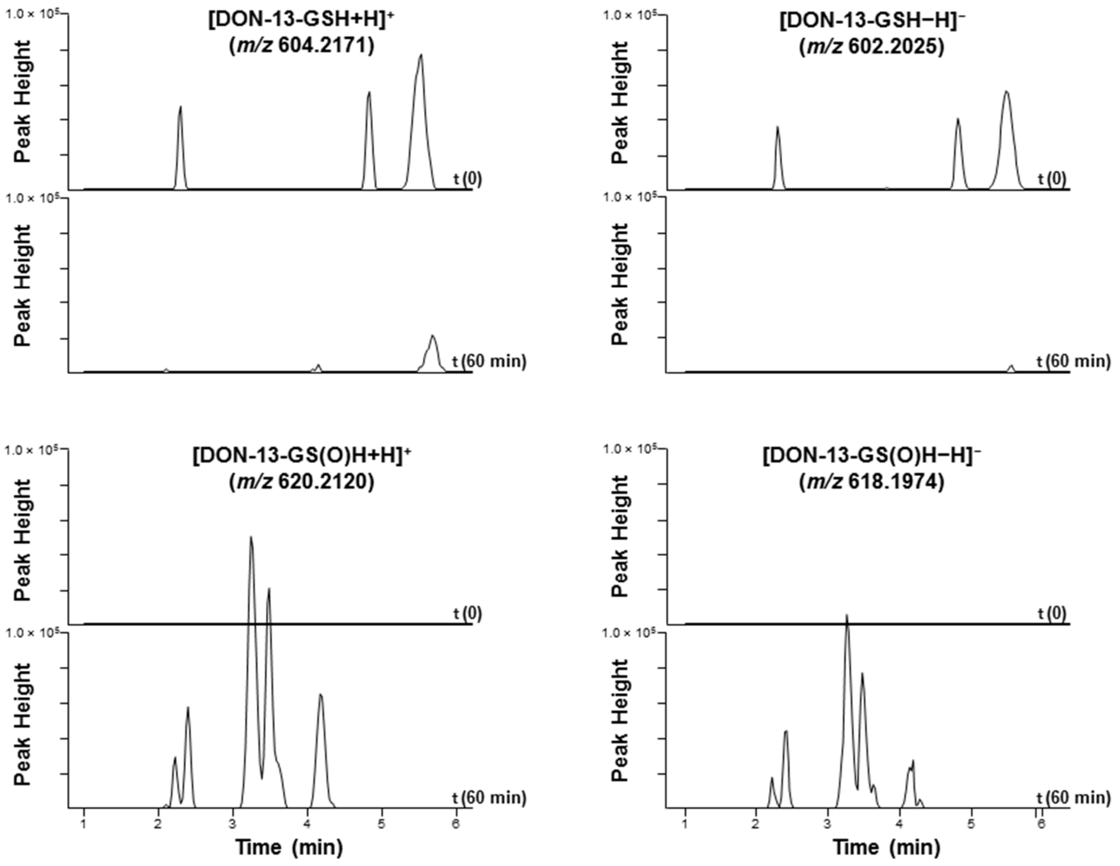

Figure S2. Extracted ion LC-HRMS chromatograms (±5 or 7.5 ppm for positive/negative ion mode, respectively) for [M + H]+ and [M − H]⁻ of a reference mixture from reaction of DON with GSH (1 week) and its corresponding sulfoxides, DON-GS(O)H. The upper two chromatograms show the disappearance of the DON-GSH peaks after treatment of the extract with hydrogen peroxide for 60 min, while the lower two chromatograms show the concurrent appearance of the partially separated peaks from pairs of DON-GS(O)H diastereoisomers. Note that the increase in peak height for the DON-GS(O)H isomers relative to the DON-GSH isomers is due to the higher injection volume (3 μL vs. 1 μL)

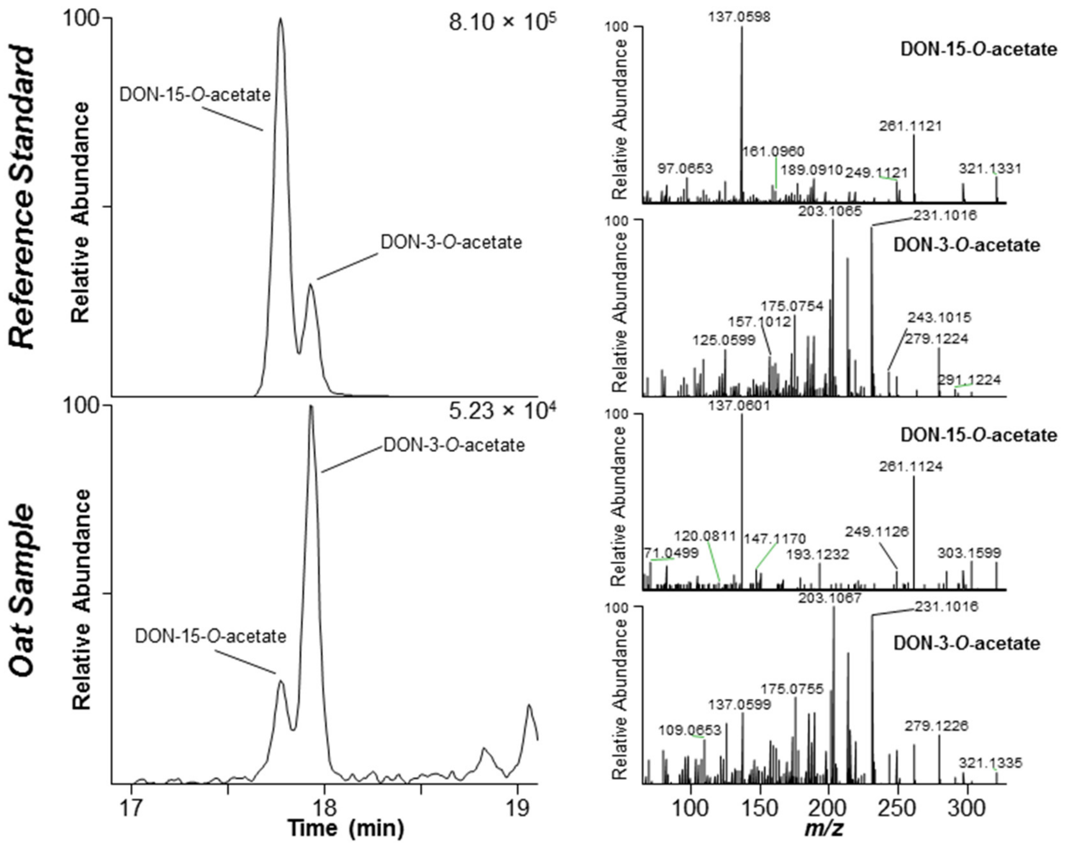

Figure S3. Extracted ion chromatograms from target-HRMS/MS and product ion spectra of the [M + H]$^+$ ions of DON-acetates in a reference standard mixture (upper traces) and an oat sample (lower traces). The number in the top right-hand corner of each chromatogram is the intensity of the highest peak in that chromatogram (arbitrary units).
